# The Immune Response against *Acinetobacter baumannii*, an Emerging Pathogen in Nosocomial Infections

**DOI:** 10.3389/fimmu.2017.00441

**Published:** 2017-04-12

**Authors:** María Guadalupe García-Patiño, Rodolfo García-Contreras, Paula Licona-Limón

**Affiliations:** ^1^Departamento de Biología Celular y del Desarrollo, Instituto de Fisiología Celular, Universidad Nacional Autónoma de México, Ciudad de México, Mexico; ^2^Facultad de Medicina, Departamento de Microbiología y Parasitología, Universidad Nacional Autónoma de México, Ciudad de México, Mexico

**Keywords:** *Acinetobacter baumannii*, neutrophil, immune response, nosocomial, resistance

## Abstract

*Acinetobacter baumannii* is the etiologic agent of a wide range of nosocomial infections, including pneumonia, bacteremia, and skin infections. Over the last 45 years, an alarming increase in the antibiotic resistance of this opportunistic microorganism has been reported, a situation that hinders effective treatments. In order to develop effective therapies against *A. baumannii* it is crucial to understand the basis of host–bacterium interactions, especially those concerning the immune response of the host. Different innate immune cells such as monocytes, macrophages, dendritic cells, and natural killer cells have been identified as important effectors in the defense against *A. baumannii*; among them, neutrophils represent a key immune cell indispensable for the control of the infection. Several immune strategies to combat *A. baumannii* have been identified such as recognition of the bacteria by immune cells through pattern recognition receptors, specifically toll-like receptors, which trigger bactericidal mechanisms including oxidative burst and cytokine and chemokine production to amplify the immune response against the pathogen. However, a complete picture of the protective immune strategies activated by this bacteria and its potential therapeutic use remains to be determined and explored.

## Introduction

*Acinetobacter baumannii* [from the Greek *akinetos bactrum*: non-motile rod; *baumannii*: after Paul Baumann (1, 2)] is a Gram-negative, non-fermenting, strictly aerobic, oxidase negative, catalase positive coccobacillus belonging to the *Moraxellaceae* family (2, 3). The importance of this bacterium relies in its ability to cause nosocomial infections (4) and its increasing antibiotic resistance (5–7). *A. baumannii* is the etiologic agent of a wide range of clinical manifestations, most frequently pneumonia, bacteremia, meningitis, urinary tract, skin and soft tissue infections; which occur preferentially among intensive care unit patients (8).

The World Health Organization has considered antibiotic resistance as one of the most serious health problems; resistance increases the length of illnesses, their morbidity and mortality, as well as their costs within health-care facilities (9). *A. baumannii* belongs to a group of bacteria termed ESKAPE, an acronym indicating *Enterococcus faecium, Staphylococcus aureus, Klebsiella pneumoniae, Acinetobacter baumannii, Pseudomonas aeruginosa*, and *Enterobacter* spp. Pathogens of this group *escape* from the effect of many antibacterial drugs and are currently the major antibiotic resistant microorganisms responsible for nosocomial infections (10, 11). Moreover, these organisms all preferentially affect immunocompromised and critically ill patients in intensive care units (12).

The presence of antibiotic resistance in *A. baumannii* complicates the implementation of effective treatments, making the development of new strategies to control the infections caused by this opportunistic microorganism mandatory. Given that bacterial clearance as well as the resolution of the infection depends not only on the effect of antibiotic drugs but also on the host’s immune response (13), it is necessary to understand how the immune system faces this pathogen. In this regard, characterization of the molecular and cellular basis of the immune response may provide the tools for the development of alternative treatments or immunotherapies against *A. baumannii*. In this review, we will summarize the current limited knowledge concerning the immune response during this infection and will discuss possible therapeutic targets to implement in future strategies to combat *A. baumannii* infections.

## Neutrophils, Essential Players During *A. baumannii* Infection

Neutrophils are essential for the control of different types of *A. baumannii* infection (14–19). An initial indication about the protective role of neutrophils was the observation of high prevalence of infections caused by *Acinetobacter* in neutropenic patients (20). In agreement, early studies characterizing the therapeutic effect of imipenem against different strains of *A. baumannii* in a mouse model had to be performed in cyclophosphamide-treated neutropenic mice, due to the low susceptibility to *A. baumannii* shown by immunocompetent mice (21). Ten years later, van Faassen and colleagues directly evaluated the neutrophil role in pneumonia caused by *A. baumannii*. They reported rapid recruitment of neutrophils at the site of infection, as early as 4 h, which peaked at 24 h postinfection. Increased lethality and severity of infection was observed in neutrophil depleted hosts, together with delayed production of cytokines and chemokines involved in neutrophil recruitment, including interleukin-1, tumor necrosis factor (TNF-α), keratinocyte chemoattractant protein (KC/CXCL1), macrophage inflammatory protein (MIP)-1, MIP-2/CXCL2, and monocyte chemoattractant protein 1 (14). These results were later confirmed by Tsuchiya and colleagues (15). In addition, the importance of early production of chemokines and proinflamatory cytokines acting on neutrophils to limit the infection was further supported by Qiu and colleagues, demonstrating that a delayed production of these molecules results in increased bacterial burdens and dissemination (16). Evidence of neutrophils limiting different types of *A. baumannii* infection including bacteremia (17), septicemia (18), and skin infection (19) has also been reported.

Neutrophils possess multiple bactericidal mechanisms, among them, the oxidative burst is the main killing defense against *A. baumannii*. At the molecular level, studies focused on the mechanisms used by neutrophils to eliminate this bacterium demonstrated a dispensable role for the nitric oxide synthase 2 and a critical requirement for the nicotinamide adenine dinucleotide phosphate phagocyte oxidase (NADPH) to kill *A. baumannii*, prevent replication and dissemination of the bacterium as well protection of the infected mice from death (22). Other novel defense mechanisms like neutrophil extracellular traps (NETs) are not induced in response to this bacterium (23), despite the fact that the formation of NETs can be stimulated through reactive oxygen species (24, 25).

The chemoattractant signals recruiting neutrophils and other cell types during *A. baumannii* infections are not limited to chemokines and some bacterial metabolites (26) as well as antimicrobial peptides produced by the host have been reported (27). Regarding the cytokines expected to be important for an efficient *A. baumannii* elimination, IL-17 has emerged as an interesting candidate given its relevance in promoting granulopoiesis and inducing cytokine, chemokine, and antimicrobial peptide expression including GM-CSF, IL-8 (a neutrophil chemoattractant and homolog human chemokine to KC and MIP-2), and LL-37, respectively (28, 29). However, despite a clear induction of IL-17A expression promoted by a peritoneal inoculation of *A. baumannii*, the neutralization of this cytokine by antibodies during septicemia, or infection in a IL-17A-deficient background, neither affected bacterial burden nor survival rate, when compared with antibody control-treated mice or wild-type (WT) mice (18). Unfortunately, during this study, the role of IL-17F [a cytokine with physiological effects shared by IL-17-A (30)] was not explored, and its involvement or even its requirement during the response to *A. baumannii* can not be excluded.

During an active *A. baumannii* infection, a small percentage of bacteria can avoid being killed by neutrophils by means of their capability to adhere to these cells independently of phagocytic processes. In this case, instead of resulting in protection, neutrophil activation can contribute to the dissemination of the bacteria, a pathogen strategy that hinders clearance and takes advantage of the migratory capacity of neutrophils. Unfortunately, the mechanisms underlying bacterial adherence to neutrophils are still unknown (31).

## Additional Innate Immune Effectors in the Immune Response Against *A. baumannii* Infection

During an *A. baumannii* infection, one of the first soluble factors produced by the host is antimicrobial peptides. Cathelicidin LL-37, whose precursor hCAP-18 (human cationic antibacterial peptide, 18 kDa) can be produced by epithelial cells and neutrophils (32), as well as some peptides naturally derived from it, present a bactericidal effect against *A. baumannii* (27). Importantly, the extent of bacterial susceptibility to LL-37 depends on the presence of lipopolysaccharide (LPS), as it has been determined that LPS-deficient mutant strains are more susceptible to the bactericidal effect of this peptide (33).

*Acinetobacter baumannii* interacts with epithelial cells through the binding of a 34-kDa protein referred as outer membrane protein A (OmpA), as well as a TonB-dependent copper receptor (an energy transducer) to fibronectin (34). One of the consequences of this interaction is the production of antimicrobial peptides. *In vitro* studies using skin and oral epithelial cells exposed to *A. baumannii* reported bacterial-induced expression of the human β-defensins (hBDs) hBD-2 and hBD-3 with antibacterial activity against *A. baumannii* (35). Interestingly, hBD-2 is also produced by airway epithelial cells during *A. baumannii* pneumonia, suggesting a conserved protective mechanism independent of the epithelial origin during an extracellular infection (36). The importance of the expression of hBDs for host protection is also observed during intracellular infections, where signaling dependent on the cytosolic pattern recognition receptors (PRRs), nucleotide-binding oligomerization domain (NOD) NOD1 and NOD2, results in hBD-2 production (37). Therefore, the use of antimicrobial peptides produced during the early stages of the infection with efficient bactericidal activity may be a therapeutic option.

The contribution of other soluble factors, like the complement system, to the control of infection has also been explored. The complement system generally contributes to limit bacterial replication (17); however, *A. baumannii* has some virulence factors that allow successful evasion of this defense mechanism (38–41). While some results point to the involvement of factor H in the evasion of the complement system by *A. baumannii* (38), others indicate otherwise (39). Factor H is one of the soluble host regulators of the alternative complement pathway, this molecule promotes the decay of the C3 convertase, C3bBb, preventing the deposition of the opsonin C3b on the host cell membranes, and acts as a cofactor for factor I, which in turn inactivates C3b (42, 43). Kim and colleagues (38) found that the OmpA, present on the surface of most *A. baumannii* strains (44), binds factor H avoiding deposition of C3b on the surface of bacteria and thus allowing evasion of the alternative complement pathway (38). However, subsequent studies by King and colleagues failed to identify the deposition of factor H on the bacterial surface. They also reported reduced C3 deposition on resistant strains compared to sensitive strains and proposed that the biofilm formation could be a mechanism through which *A. baumannii* evade the complement system. This discrepancy suggests that different strains of *A. baumannii* could use different strategies to circumvent complement-mediated killing.

Additional virulence factors produced by *A. baumannii*, such as CipA and PKF, are also implicated in the evasion of the complement system (40, 41). CipA, an outer membrane protein, binds to the active form of plasminogen, plasmin, to degrade fibrinogen and promote bacterial dissemination. This CipA–plasmin complex also degrades C3b; however, there is no correlation between the levels of CipA–plasmin and complement resistance so far. Hence, the mechanism through which CipA confers complement resistance still needs to be elucidated (40). PKF, a secreted serine protease, could also have a role in the cleavage of some complement components; nevertheless, the complement components susceptible for the action of this protein remain to be identified. In conclusion, several mechanisms have been proposed to explain complement evasion by *A. baumannii*, all centered in avoiding C3b deposition at the bacterial surface, which then decreases opsonization and subsequent phagocytosis, declines the C5 convertase and C3bBbC3b formation (45), to finally prevent the membrane attack complex formation (46) and promote bacterial survival.

Besides the essential role of neutrophils in resolving *A. baumannii* infections, other immune cell types have been shown to be activated in response to this opportunistic pathogen. Monocytes and macrophages are among the first responding cells to be recruited and/or activated by *A. baumannii*. Tissue-resident macrophages, such as alveolar macrophages, would be present at the site of infection before the recruitment of neutrophils. This situation confers an advantage for the early response against *A. baumannii*, so that macrophages can phagocyte and limit bacteria while neutrophils are recruited. *In vivo*, phagocytosis of *A. baumannii* by macrophages can be observed as early as 4 h postinfection, by then, few neutrophils have been recruited, and the former cells have phagocytosed only a small amount of bacteria. Phagocytosis by macrophages *in vitro* can be detected as soon as 10 min after macrophage interaction with *A. baumannii* (47).

In addition to phagocytosis, macrophages produce high amounts of MIP-2, IL-6, and TNF-α in response to *A. baumannii* infection. Early production of MIP-2 by macrophages might be relevant for neutrophil recruitment but has not been formally proven. At extended periods postinfection (approximately 48 h), high levels of the previously mentioned cytokines and chemokines are maintained by macrophages, together with an increment in the production of other cytokines, including IL-10 and IL-1β. Even though macrophages take longer to kill equivalent amounts of bacteria than neutrophils do, the first ones are capable of killing more than 80% of the phagocytosed bacteria within the first 24 h. A confirmed mechanism used by macrophages to kill bacteria is the production of nitric oxide (47). Depletion of macrophages in an *in vivo* model of pneumonia resulted in a higher bacterial burden in comparison with control mice; however, unlike depletion of neutrophils (14), the lack of macrophages does not increase infection lethality (15, 47). Similar results, showing an increased bacterial burden, were observed in a bacteremia model where macrophages were also depleted (17). These findings suggest that macrophages may be dispensable for the resolution of *A. baumanni*i infection, but they might help to control bacterial replication at early phases of the pathogen–host interaction.

Natural killer cells (NKs) represent another immune cell type acting during the early defense response against *A. baumannii*. Depletion of NKs in a pneumonia model interferes with bacterial clearance and hence resolution of the infection. The mechanism through which NKs contribute to control *A. baumannii* pneumonia is indirect and relies on the production of the chemoattractant KC, which in turn recruits neutrophils to the site of infection (15).

Finally, dendritic cells (DCs), the bridge between innate and adaptive immune responses, have been shown to become activated in response to *A. baumannii* LPS. Moreover, OmpA activates DCs’ signaling *via* mitogen-activated protein kinases (MAPKs) and nuclear factor kappa B (NFκB), thus resulting in high expression of molecules involved in antigen presentation and production of the inflammatory cytokine IL-12. As a consequence, DCs are prone to polarize T cells into TH1 effectors (48).

## Cell Receptors Involved in the Recognition of *A. baumannii*

The activation of immune cells largely depends on its ability to recognize pattern-associated molecular patterns (PAMPs) through PRRs. Similar to other infections (49), two groups of PRRs, toll-like receptors (TLRs) and NOD receptors, have been implicated in the recognition of *A. baumannii*. While the role of TLR-2 and TLR-4 (two main TLRs recognizing PAMPs during bacterial infections) (50) has been widely explored in the context of *A. baumannii* infection (51–53), little is known about the recognition of *A. baumannii* through NOD receptors. A possible explanation is that *A. baumannii* is mainly considered an extracellular pathogen; however, as previously discussed, there are reports describing epithelial NOD1 and NOD2 activation by *A. baumannii* (37). Among the cell types recognizing PAMPs present in *A. baumannii* through TLRs, monocytes, macrophages, epithelial cells, and DCs have been identified (33, 36, 48, 54). In addition, neutrophils are able to recognize *A. baumannii* through TLR-2 and TLR-4 (55), and potentially other TLRs expressed in these cells could be important; however, solid evidence demonstrating this is still missing.

Lipopolysaccharide, one of the main immunogenic molecules present in most bacteria, is a well-established ligand for TLR-4 (56) and probably the most-studied virulence factor from *A. baumannii*. During *A. baumannii* infection, TLR-4 along with CD14 [a glycosylphosphatidylinositol-linked membrane protein that allows LPS recognition through TLR-4 (57)] contribute to the recognition and later resolution of infection, as demonstrated by Knapp and colleagues. Experiments performed in TLR-4- and CD14-deficient mice demonstrated the importance of these molecules in the recognition and clearance of *A baumannii* as higher bacterial burdens, and a higher degree of bacterial dissemination was observed in comparison to WT mice during a model of pulmonary infection. Furthermore, the TLR-4-deficient mice showed a decrease in the polymorphonuclear cell recruitment to the lungs, thus resulting in diminished inflammation. The inability of these mice to control bacterial replication and dissemination is directly correlated with low TNF-α, IL-6, MIP-2, and KC production (51).

Interestingly, it has been reported that LPS from different *A. baumannii* strains is mitogenic for splenic cells and induces the production of IL-8 in the monocytic-like cell line THP-1 as well as TNF-α in splenic and THP-1 cells (54, 58). The role of TLR-4 in the recognition of *A. baumannii* was assessed through stimulation with different clinical isolates in HEK-293 cells (an epithelial TLR-deficient cell line), transfected to induce the expression of TLR-4 and MD2 [a protein that associates with TLR-4 to allow for the recognition of LPS (59)]. This stimulation resulted in cell signaling by activation of NF-kB-induced transcription (54). A recent study, recognized TLR-4 as a key player during the immune response against *A. baumannii* demonstrating that the recognition of the bacteria through TLR-4 promotes a signal dependent on MAPKs and activation of NF-kB, both essential for the production of efficient levels of IL-6, IL-12, and TNF-α by macrophages and DCs. Signaling through TLR-4 is also necessary for the production of bactericidal NO by macrophages, the main bactericidal strategy against *A. baumannii* identified so far (52).

The role of TLR-2, a receptor involved in the recognition of peptidoglycan and lipoproteins (60), during the response against *A. baumannii*, remains controversial. Initial studies suggested that TLR-2 could contribute to *A. baumannii* recognition. In these studies, stimulation of TLR-4-deficient macrophages with a LPS-deficient strain induced production of TNF-α, while stimulation of TLR-2-deficient macrophages with the same strain was unable to induce this cytokine, indicating that *A. baumannii* possess PAMPs different than LPS that could be recognized by TLR-2, and thus suggesting that this receptor could be involved in *A. baumannii* recognition (33). Currently, one of the PAMPs recognized by TLR-2 in *A. baumannii* is OmpA, whose effects on DCs were described above (48). Supporting the defensive role of TLR-2 during *A. baumannii* infection, stimulation of HEK-293 cells transfected with a TLR-2/CD14 construct, by whole inactivated *A. baumannii* strains, induced signaling through TLR-2 (54). Additional evidence about the contribution of TLR-2 in the resolution of infection was given by March and colleagues, showing that the production of IL-8 by the epithelial cell line A549 was promoted through the recognition of *A. baumannii* by both TLR-4 and TLR-2 (36). Similarly, Kim and colleagues reported that TLR-2 contributes to the pulmonary clearance of *A. baumannii* (53). On the other hand, Knapp and colleagues found that TLR-2-deficient mice had lower bacterial burden at early stages of pulmonary infection as well as earlier polymorphonuclear cell recruitment to the lung when compared to WT mice (51). However, other results generated by Kim and colleagues failed to support previous findings on the role of TLR-2 in response to *A. baumannii* infection (52). These discrepancies could be due to the use of different *A. baumannii* strains, the infection model, dose and administration routes, or even due to the cellular type used in each study (see Table 1).

**Table 1:** **Research models used to study host responses against *Acinetobacter baumannii***.

*A. baumannii* strain	Approach	Model/experimental design	Major findings	Reference
ATCC 17961	*In vivo*	Bronchopneumonia	Neutropenia increases *A. baumannii* susceptibility as a result of delayed production of cytokines and chemokines	(14)
		1 × 10^7^ CFU intranasally inoculated in 8–12 weeks old female C57BL/6 and BALB/c mice		

A112-II-a (nephritis clinical isolate)	*In vivo*	Bronchopneumonia	Natural killer cells recruit neutrophils through KC production	(15)
		1 × 10^7^–1 × 10^8^ CFU intranasally inoculated in 8–10 weeks old female C57BL/6 mice		

ATCC 17961	*In vivo*	Bronchopneumonia	Delayed and reduced production of chemokines and cytokines promote severe bronchopneumonia	(16)
		Intranasally inoculated 8–12 weeks old A/J and C57BL/6 female mice with 1 × 10^7^–1 × 10^8^ CFU		

ATCC and clinical isolates: HUMC1, LAC-4, HUMAC4, HUMC5, HUMC6, C14, AB0061, AB0068, UH7807, 17978, R2, 31 (clone B), 125, 152 (clone A), AB0071, AB0072, AB0074, AB0093, METRO 9, UH2207, UH4907, UH5107, UH5207, UH6507, UH7007, UH7507, UH8107, UH8307, UH8407, UH9007, UH9707, AB7075	*In vivo/in vitro*	*In vivo*: bacteremia. Intravenously infected C3H/FeJ mice with 1.5 × 10^7^–8 × 10^8^ CFU	Complement system, macrophages, and neutrophils are involved in the defense mechanisms against *A. baumannii*	(17)
		*In vitro*: complement susceptibility in BALB/cJ mouse serum. Phagocytosis by the murine macrophage cell line RAW264.7		

576, 4502, 5798, 6143, and 7215 clinical isolates	*In vivo*	Septicemia. Intraperitoneally inoculated 6–8 weeks old C57BL/6J, C3HeB/FeJ, and IL-17a^−/−^ knockout mice with 2.15 × 10^6^–9.2 × 10^6^ CFU	Dispensable role for IL-17A to control *A. baumannii* septicemia	(18)

0057, 1422, 1611, 2098, 2231, 3559, and 7405 clinical isolates	*In vivo*	Wound infection	Neutropenia causes a more severe *A. baumannii* wound infection	(19)
		Wound inoculated 6–8 weeks old BALB/c mice with 1 × 10^7^ CFU		

ATCC 17961	*In vivo*	Bronchopneumonia. Intranasally inoculated 8–12 weeks old B6.129S-Cybb^tm1⋅Din^/J (NADPH oxidase-deficient [gp91^phox−/−^]), B6.129P2-Nos2^tm1⋅Lau^/J (inducible nitric oxide synthase-deficient [NOS2^−/−^]), and C57BL/6 female mice with 1 × 10^7^ CFU	Indispensable role for the NADPH phagocyte oxidase to control replication and dissemination of *A. baumannii*	(22)

ATCC 19606	*In vitro*	Human blood neutrophils in the presence of 5 × 10^7^ CFU	*A. baumannii* infection does not induce neutrophil extracellular traps formation	(23)

ATCC 17978, ATCC 17978::GFP, 17978ΔgacS, 17978 pgacS, 17978ΔgacA, 17978 pgacA, 17978ΔpaaA, 17978 ppaa, 17978ΔcsuD, M2, M2ΔabaI, and M2 pabaI mutant	*In vivo*	Septicemia	The bacterial metabolite phenylacetate is chemotactic for neutrophils during *A. baumannii* infection	(26)
		Intravenously infected zebra fish embryo with 1 × 10^3^ CFU		
		Intraperitoneally infected 6–8 weeks old BALB/c female mice with 5 × 10^4^ CFU		

ATCC 19606™ and AB5075, AB5711, AB#4, and AB4795 clinical isolates	*In vitro*	*A. baumannii* culture in the presence of LL-37 and its derived peptides	Bactericidal activity of LL-37 against *A. baumannii*	(27)

ATCC 19606	*In vivo/in vitro*	Bronchopneumonia	*A. baumannii* adheres to neutrophils to spread in the host and avoid bactericidal mechanisms of neutrophils	(31)
		Intratracheally infected 6 weeks old C3H/HeN female mice with 5 × 10^7^ CFU		
		*In vitro*: adherence, transmigration assays, and cytokine production in human blood neutrophils cultured in the presence of bacteria		

ATCC 19606 and 19606R [lipopolysaccharide (LPS)-deficient mutant]	*In vitro*	Murine macrophage cell line RAW264.7 and immortalized toll-like receptor (TLR)-2-deficient, TLR-4-deficient, and MyD88/Mal-deficient murine macrophages in the presence of bacteria	Increased susceptibility to LL-37 in LPS-deficient *A. baumannii*	(33)
			Recognition of *A. baumannii* through TLR-2	

AB0057 and ATCC 17978 isolates	*In vitro*	Primary cultures of oral or skin epithelial cells in the presence of *A. baumannii* (MOI 100)	Induction of hBDs, hBD-2 and hBD-3 in epithelial cells as a response to *A. baumannii*	(35)

1514, 670, 1064, and 1327 clinical isolates	*In vitro*	*A. baumannii* (MOI 100) incubated with human lung epithelial cell line A549 (ATCC CCL185) or primary human airway epithelial cells	Involvement of TLR-2 and TLR-4 in *A. baumannii* recognition and IL-8 production *via* NFκB and mitogen-activated protein kinases	(36)
			Induction of hBD-2 in response to bacteria	

ATCC 19606™	*In vitro*	Human lung epithelial cell line A549 (ATCC CCL185), Nod1-, Nod2-, or Rip2-knocked down THP-1-derived macrophages or NFκB-luciferase/hBD-2-luciferase expressing HEK293T cell line in the presence of *A. baumannii* (MOI 100)	*A. baumannii* recognition through nucleotide-binding oligomerization domain (NOD)1 and NOD2 and hBD-2-mediated bacterial clearance	(37)

DAB021, KA10, 04P412, and 05KA010 clinical isolates	*In vitro*	*A. baumannii* incubated with human serum	Evasion of complement system through Omps-factor H binding	(38)

LK10, LK15, LK18, LK41, LK49, LK80, and LK88 clinical isolates	*In vitro*	*A. baumannii* incubated with human serum	Ability of *A. baumannii* to be recognized by alternative complement pathway	(39)

ATCC 19606, ATCC 17978, and 11CS, 15CS, 17CS, 25CS, 27CS, V754948 clinical isolates, and ΔcipA mutant	*In vitro*	*A. baumannii* incubated with human serum	Complement system evasion through CipA degradation of C3b	(40)
		Human umbilical vein endothelial cell line cocultured with bacteria (MOI 100)		

LK10, LK41, and LK88 clinical isolates, and LK41.3 (PKF-deficient mutant)	*In vitro*	*A. baumannii* incubated with human serum	Role of PKF in complement system evasion	(41)

ATCC 17961	*In vivo/in vitro*	Bronchopneumonia. Intranasally inoculated 8–12 weeks old BALB/c mice with 1 × 10^8^ CFU	Role of macrophages in early stages of *A. baumannii* bronchopneumonia	(47)
		*In vitro*: phagocytosis, bactericidal assay, and cytokine/chemokine production using the monocyte–macrophage J774A.1 cell line (ATCC TIB-67, J774) in the presence of *A. baumannii* (MOI 100)		

ATCC 19606™	*In vitro*	Outer membrane protein A (OmpA)-stimulated bone marrow derived-dendritic cells (DCs)	OmpA from *A. baumannii* induces DC activation and confers them the ability to polarize T CD4^+^ cells toward a TH1 phenotype	(48)

RUH 2037 (pneumonia clinical isolate)	*In vivo*	Bronchopneumonia	Description of TLR-4 and CD14 in the control of *A. baumannii* pneumonia	(51)
		1 × 10^6^–1 × 10^8^ CFU intranasally inoculated in 7–9 weeks old C57/BL6, CD14^−/−^, TLR-4^−/−^, and TLR-2^−/−^ mice		

KCCM 35453 (ATCC 15150)	*In vitro*	Wild-type, TLR-2^−/−^, and TLR-4^−/−^ bone marrow-derived macrophages and DCs cocultured with different MOI of bacteria	TLR-4-mediated cytokine and nitric oxide production in response to *A. baumannii*	(52)

KCCM 35453 (ATCC 15150)	*In vivo*	Bronchopneumonia. Intranasally inoculated C57/BL6 and TLR-2^−/−^ mice with 3 × 10^7 ^CFU	TLR-2 limits *A. baumannii* replication at early stages of pneumonia	(53)

4801, 4802, 4803, 4808, and 4809 clinical isolates	*In vitro*	*A. baumannii*-stimulated human monocyte THP-1, and human embryonic kidney HEK-293 TLR-2- and TLR-4-expressing cell lines	LPS from *A. baumannii* induces tumor necrosis factor (TNF-α) production in THP-1 cells	(54)
			UV killed *A. baumannii* is recognized by TLR-2 and TLR-4	

HS-54, HJJA-9, HJJA-7, UC-25, HS-4 HJJA-8, and 95-52 clinical isolates	*In vitro*	*A. baumannii*-derived LPS incubated with splenic cells from 6 to 8 weeks old BALB/c mice	TNF-α induction and mitogenic capacity of LPS from *A. baumannii*	(58)

ATCC 17978, HUMC1, HUMC4, HUMC5, HUMC6, and HUMC12	*In vivo*	Septicemia	Induction of protective anti-*A. baumannii* and anti-OmpA antibodies by active and passive immunization with recombinant protein	(63)
		Intravenous infection with 1 × 10–2 × 10^7^ CFU in 10 weeks to >6 months old BALB/c streptozotocin-induced diabetic mice	Increased phagocytosis by specific anti-OmpA antibodies	
		Immunization. Subcutaneous administration of 3 µg of recombinant OmpA plus Al(OH)_3_ to BALB/c mice or passive immunization with immune serum		

None (recombinant OmpA)	*In vivo*	Subcutaneous administration of 3, 30, or 100 µg of recombinant OmpA plus Al(OH)_3_ to BALB/c mice	Predominance of IgG1 antibody subtype and activation of IFN-γ, IL-4, and IL-17 producing splenocytes after immunization with OmpA	(64)
			Induction of IFN-γ/IL-4 or IL-4 cytokine profile depending on the dose of antigen (OmpA) during immunization	

## Adaptive Immune Response Against *A. baumannii*

Despite the lack of information concerning the contribution of cells from the adaptive immune system in the control and resolution of *A. baumannii* infections, humoral immunity has been extensive explored in an attempt to design an effective and safe vaccine. Many bacterial antigens have been proposed as candidates for a vaccine development (61, 62). Currently, OmpA emerges as one of the best, given its high immunogenicity in mice and in humans (63–65) as well as its broad distribution as a virulent factor among many different *A. baumannii* strains (44, 63). The induction of specific anti-*A. baumannii* antibodies during infection has been reported, illustrating OmpA is a major antigen able to promote a humoral antibody response. Using a diabetic mouse model, previously shown to be a susceptible host for *A. baumannii* infection (66), Luo and colleagues demonstrated that active and passive immunization with OmpA confers protection against *A. baumannii*. Furthermore, it was confirmed that one of the mechanisms through which specific anti-OmpA antibodies exert protection is by bacterial opsonization, leading to an increment in macrophage-mediated phagocytosis. The same report also suggested that the conferred protection was independent of complement activation (63). Immunization with recombinant OmpA not only results in the production of specific IgG1 antibodies (induced during TH2-type responses) and activation of IFN-γ-, IL-4-, and IL-17-producing splenocytes in an antigen-specific manner but also depending on the antigen dose and immunization results in the production of different cytokine profiles. Thus employing low doses (3 µg) of this antigen, an IFN-γ/IL-4 profile is reached, while immunization with higher doses of recombinant OmpA (100 µg) induces an IL-4 profile, characteristic of TH2 responses (64).

## Conclusion

Altogether, cumulative evidence of the host response against *A. baumannii* demonstrates the participation of several immune cell types including monocytes, macrophages, and DCs in the control and resolution of the infection, with an essential role for neutrophils; however, the use of immunotherapies has been largely ignored. Considering that neutrophils are the main immune cell population preventing *A. baumannii* infections, it is attractive to consider the development of immunotherapies based on the use of cytokines and chemokines acting on neutrophil recruitment and activation such as MIP-2 and KC.

Current information about the immune response against the infection caused by *A. baumannii* has been generated from different studies that focused on the role of just a few cellular types at once, as well as on studies that explore the immunogenic effects of a single pathogen structure on a specific cell type. However, a deeper understanding that provides a more complete vision of the global immune response taking place during *A. baumannii* infection as well as additional studies focusing on the kinetics of this response is mandatory.

**Figure 1:**
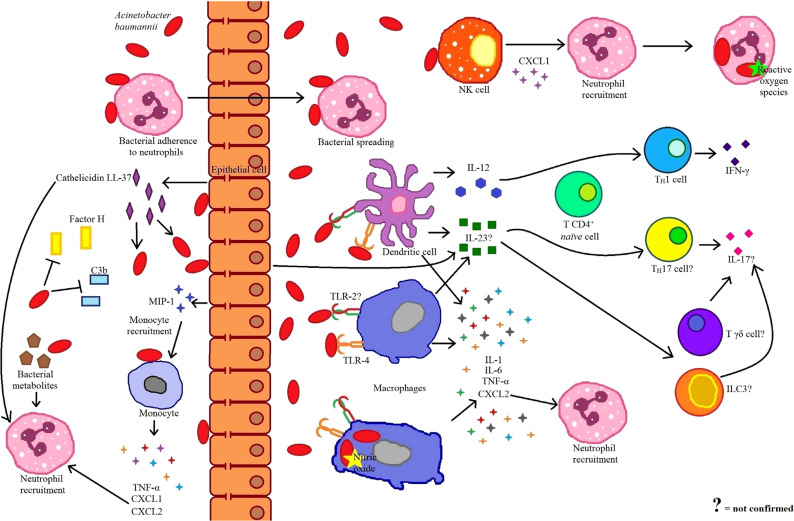
**Immune response to *Acinetobacter baumannii* infection**. Antimicrobial peptides, produced by epithelial cells are one of the first bactericidal mechanisms against *A. baumannii*. At the same time, these antimicrobial peptides act as chemoattractants for neutrophils. *A. baumannii* possesses evasion mechanisms to avoid deposition of complement system components such as factor H and C3b. Epithelial cells recognize bacteria and secrete macrophage inflammatory protein 1 (MIP-1) to recruit monocytes. In turn, these monocytes, respond to *A. baumannii* secreting tumor necrosis factor (TNF-α), CXCL1, and CXCL2 to recruit neutrophils. A small percentage of bacteria evade neutrophil phagocytosis by adhering to the neutrophil surface and exploiting the migratory ability of these cells to disperse through the host. The chemokines CXCL1 and CXCL2, secreted in response to bacteria, as well as bacterial metabolites, serve as chemotactic factors for neutrophils. Once bacteria crossed through the epithelium, they can be recognized by natural killer (NK) cells, which respond by secreting CXCL1 and recruiting more neutrophils. Other innate immune cells, such as macrophages and dendritic cells (DCs), also recognize bacteria through toll-like receptor (TLR)-4 and TLR-2. Both DCs and macrophages produce proinflamatory cytokines in response to *A. baumannii*, and while macrophages secrete CXCL2 to recruit neutrophils, DCs process and present the bacteria to CD4^+^ T *naïve* cells polarizing toward a TH1 profile. The main mechanism through which *A. baumannii* infection can be controlled by macrophages is by the bactericidal effect of nitric oxide; while neutrophils kill *A. baumannii* by the production of reactive oxygen species. Because of its importance in responses that involve neutrophils, it has been considered, but not confirmed, the participation of IL-17 during *A. baumannii* infections. This cytokine could be produced by different cells including TH17, Tδγ, and type 3 innate lymphoid cells (ILC3), all induced in the presence of IL-23 secreted by macrophages, DCs, and epithelial cells.

Most of the information about the host response against *A. baumannii* refers to the innate immune response. In fact, given the short period that it takes for the resolution of the infection (51), it has been proposed that innate immunity is enough to control *A. baumannii* (15) (Figure 1). Nevertheless, the involvement of the adaptive immune response in the control and protection against *A. baumannii* should not been ignored. Currently, there is no evidence for the requirement of the T cell adaptive immune response in the control of the infection; however, antibody induction has been considered as a prophylactic treatment (67). Characterization of the role of other immune cell populations in the defense against *A. baumannii* is still missing, particularly those that can interact with neutrophils (68), such as invariant natural killer T cells, γδ T cells, and innate lymphoid cells. In addition, most of the evidence so far focuses on the immune response against *A. baumannii* in pneumonia models or by *in vitro* stimulation, a situation that hampers the analysis between different cell types and possible interactions that may be essential for an efficient protective response. Given the increasing concern of *A. baumannii* infection as a relevant pathogen in nosocomial infections, as well as its alarming capacity to develop antibiotic resistance, in the future it would be important to perform additional studies focusing on the immune response observed in different types of infection by this bacteria, in order to develop alternative strategies to ensure an efficient clearance and survival of the host.

## Author Contributions

MG-P wrote and discussed the review, PL-L and RG-C conceived the idea, and PL-L discussed and revised the manuscript.

## Conflict of Interest Statement

The authors declare that the research was conducted in the absence of any commercial or financial relationships that could be construed as a potential conflict of interest.
