# Corrigendum: The Immune Response Against *Acinetobacter baumannii*, an Emerging Pathogen in Nosocomial Infections

**DOI:** 10.3389/fimmu.2022.960356

**Published:** 2022-06-27

**Authors:** María Guadalupe García-Patiño, Rodolfo García-Contreras, Paula Licona-Limón

**Affiliations:** ^1^ Departamento de Biología Celular y del Desarrollo, Instituto de Fisiología Celular, Universidad Nacional Autónoma de México, Ciudad de México, Mexico; ^2^ Facultad de Medicina, Departamento de Microbiología y Parasitología, Universidad Nacional Autónoma de México, Ciudad de México, Mexico

**Keywords:** *Acinetobacter baumannii*, neutrophil, immune response, nosocomial, resistance

In the published article, there was an error. The PhD program of the first author requires a statement to be included in the review, in order for her to obtain her PhD degree.

A correction has been made to **Acknowledgements**. This sentence previously stated:

“We apologize to the researches whose work could not be cited due to space limitations. We also thank J. L. Ramos-Balderas for technical assistance and T. K. Wood, T. Rosenbaum, M. Furlan- Magaril, and J. Henao-Mejiía for critical reading of the manuscript.”

The corrected Acknowledgments appears below:

“We apologize to the researchers whose work could not be cited due to space limitations. We also thank Ramos-Balderas JL for technical assistance and Wood TK, Rosenbaum T, Furlan-Magaril M and Henao-Mejía J for critical reading of the manuscript. MGG-P is a doctoral student from the Programa de Doctorado en Ciencias Biomédicas, Universidad Nacional Autónoma de México and has received CONACYT fellowship 288933.”

The authors apologize for this error and state that this does not change the scientific conclusions of the article in any way. The original article has been updated.

## Publisher’s Note

All claims expressed in this article are solely those of the authors and do not necessarily represent those of their affiliated organizations, or those of the publisher, the editors and the reviewers. Any product that may be evaluated in this article, or claim that may be made by its manufacturer, is not guaranteed or endorsed by the publisher.

